# PROMOTING EFFECTIVE CAREER DEVELOPMENT IN GERONTOLOGY THROUGH APPLIED INTERGENERATIONAL PROGRAMS

**DOI:** 10.1093/geroni/igz038.2099

**Published:** 2019-11-08

**Authors:** Margaret M Manoogian

**Affiliations:** Western Oregon University, Monmouth, Oregon, United States

## Abstract

With the rise in global older adult populations, university programs need to produce an effective, gerontology-trained workforce (Silverstein & Fitzgerald, 2017). Career decision-making involves interactive learning (Super, 1990), as adults explore career options, engage in career learning, and understand curriculum integration within professional settings (Savickas, 2013). Gerontology faculty can utilize career planning models that integrate intergenerational engagement within the curriculum to aid student career decisions (Reardon, Lenz, Peterson, & Sampson, 2012). This paper provides an overview of a career planning model and highlights the ways intergenerational programs can be intentionally staged in research, service, and extracurricular domains to promote career planning and success in post-graduate employment. Data from our recent gerontology alumni survey including graduates since the program inception will be outlined to support the importance and success of developing strong applied intergenerational career programs in gerontology.

